# Ethyl 2-(4-meth­oxy­phen­yl)-6-oxa-3-aza­bicyclo[3.1.0]hexane-3-carboxyl­ate: crystal structure and Hirshfeld analysis

**DOI:** 10.1107/S2056989017009987

**Published:** 2017-07-21

**Authors:** Julio Zukerman-Schpector, Fabricia H. Sugiyama, Ariel L. L. Garcia, Carlos Roque D. Correia, Mukesh M. Jotani, Edward R. T. Tiekink

**Affiliations:** a Departmento de Química, Universidade Federal de São Carlos, 13565-905 São Carlos, São Paulo, Brazil; b Instituto de Química, Universidade Estadual de Campinas, UNICAMP, CP 6154, 13084-971, Campinas, São Paulo, Brazil; c Department of Physics, Bhavan’s Sheth R. A. College of Science, Ahmedabad, Gujarat 380001, India; d Centre for Crystalline Materials, School of Science and Technology, Sunway University, 47500 Bandar Sunway, Selangor Darul Ehsan, Malaysia

**Keywords:** crystal structure, pyrrolid­yl, epoxide, Hirshfeld surface analysis

## Abstract

The title compound, C_14_H_17_NO_4_, features an epoxide-O atom fused to a pyrrolidyl ring, the latter having an envelope conformation with the N atom being the flap. The 4-meth­oxy­phenyl group is orthogonal to [dihedral angle = 85.02 (6)°] and lies to the opposite side of the five-membered ring to the epoxide O atom, while the N-bound ethyl ester group (r.m.s. deviation of the five fitted atoms = 0.0187 Å) is twisted with respect to the ring [dihedral angle = 17.23 (9)°]. The most prominent inter­actions in the crystal are of the type methine-C—H⋯O(carbon­yl) and these lead to the formation of linear supra­molecular chains along the *c* axis; weak benzene-C—H⋯O(epoxide) and methine-C—H⋯O(meth­oxy) inter­actions connect these into a three-dimensional architecture. The analysis of the Hirshfeld surface confirms the presence of C—H⋯O inter­actions in the crystal, but also the dominance of H⋯H dispersion contacts.

## Abstract 2

In the title compound, the epoxide O atom and the 4-meth­oxy­phenyl group lie on opposite sides of the pyrrolidyl ring, whereas the ethyl ester is approximately planar. Linear supra­molecular chains sustained by methine-C—H⋯O(carbon­yl) inter­actions are evident in the mol­ecular packing.

## Chemical context   

α-Glucosidase inhibitors have shown potential for the treatment of several health conditions such as cystic fibrosis, diabetes, influenza and cancer. In this context, a thorough patent review on α-glucosidase inhibitors was published recently (Brás *et al.*, 2014 ▸). Among α-glucosidase inhibitors are a series of natural products including amino­ciclitols (I) and (II); see Scheme 1. The tri-hydroxyl-substituted compound (I) is found in several plants, *e.g. Morus alba* (Asano *et al.*, 1994 ▸), *Arachniodes standishii* (Furukawa *et al.*, 1985 ▸), *Angylocalyx boutiqueanus* (Nash *et al.*, 1985*a* ▸), *Hyacinthoides non-scripta* (Watson *et al.*, 1997 ▸) among others, whereas the di-hydroxyl substituted compound (II) is found in the seeds of *Castanospermum austral* (Nash *et al.*, 1985*b* ▸).

In a search for an effective synthetic path, *e.g*. good yield, to obtain both (I) and (II), it was found that they could be prepared starting from a common epoxide inter­mediate (III), which in turn could be prepared (Garcia, 2008 ▸) from (IV) when subjected to a Prilezhaev epoxidation (Prilezhaev, 1909 ▸; Swern, 1949 ▸). Herein, the crystal and mol­ecular structures of (III) are described, motivated by the desire to unambiguously establish the relative configuration of the stereogenic centres. A further evaluation of the supra­molecular association has been undertaken by analysing the Hirshfeld surface of (III).
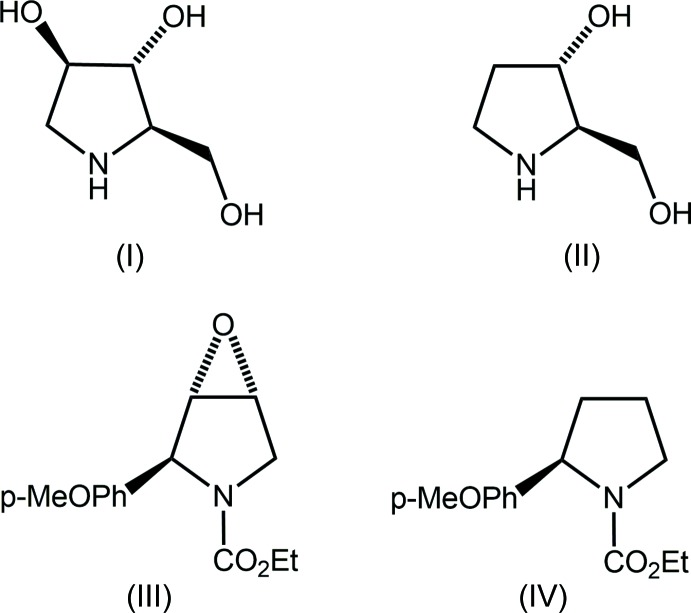


## Structural commentary   

**Figure 1:**
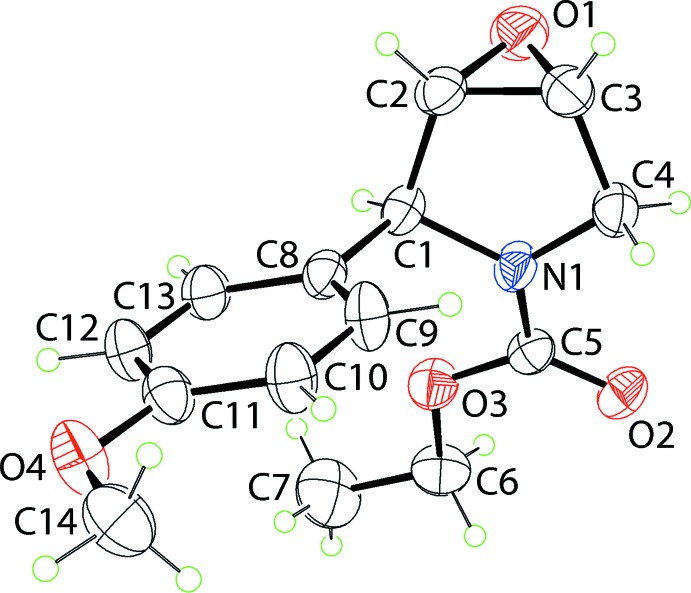
The mol­ecular structure of (III), showing the atom-labelling scheme and displacement ellipsoids at the 35% probability level.

The mol­ecular structure of (III), Fig. 1 ▸, comprises a pyrrolidyl ring fused to an epoxide O1 atom giving rise to a locally (mirror) symmetric fused-ring system. The nitro­gen atom is connected to an ethyl ester group, with the carbonyl-O2 atom orientated towards the ring-methyl­ene group. The pyrrolidyl ring is substituted in a 2-position by the 4-meth­oxy­phenyl group. The conformation of the pyrrolidyl ring is an envelope with atom N1 being the flap atom and occupying a position *syn* to the epoxide-O1 atom. The dihedral angle between the fused three- and five-membered rings is 78.53 (10)°, indicating an almost orthogonal relationship. To a first approximation, the ethyl carboxyl­ate group (r.m.s. deviation of the five non-hydrogen atoms = 0.0187 Å) is planar and forms a dihedral angle of 17.23 (9)° with the five-membered ring. The 4-meth­oxy­phenyl substituent is also approximately planar with an r.m.s. deviation of 0.0274 Å for the eight fitted non-hydrogen atoms; the small twist of the meth­oxy group out of the plane of the benzene ring to which is connected, *i.e*. the C14—O4—C11—C12 torsion angle is 175.67 (18)°, is primarily responsible for the deviations from exact planarity. The orthogonal relationship between this plane and that through the pyrrolidyl ring is seen in the dihedral angle formed between them of 85.02 (6)°. Globally, the mol­ecule has an extended planar region, comprising the pyrrolidyl ring and the ethyl ester residue with the epoxide O atom lying to one side of this plane and the 4-meth­oxy­phenyl substituent to the other.

The chirality of each of the methine-C1–C3 atoms in the mol­ecule illustrated in Fig. 1 ▸, is *S*, *S* and *R*, respectively, with the centrosymmetric unit cell containing equal amounts of both enanti­omers.

## Supra­molecular features   

**Figure 2:**
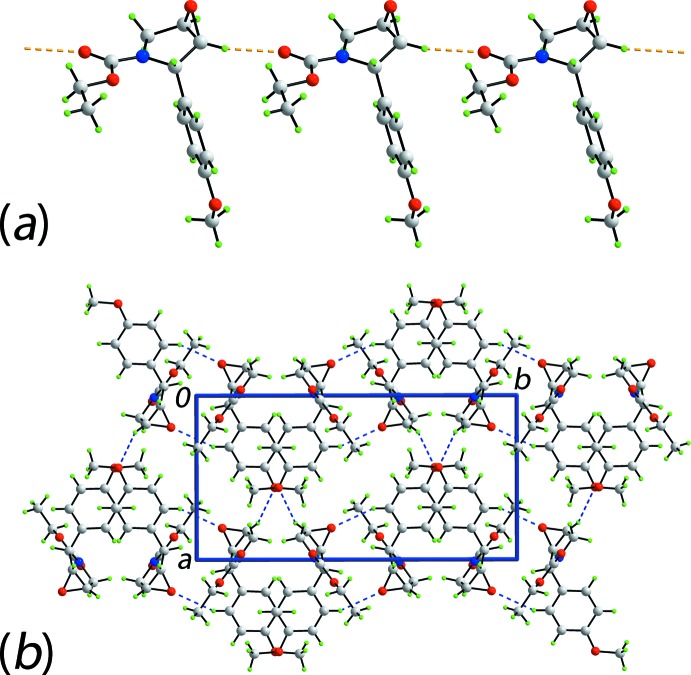
The mol­ecular packing in (III): (*a*) a view of the supra­molecular chain sustained by methine-C—H⋯O(carbon­yl) inter­actions shown as orange dashed lines and (b) a view of the unit-cell contents in projection down the c axis, whereby the chains illustrated in (a) are linked by weak benzene-C—H⋯O(epoxide) and methine-C—H⋯O(meth­oxy) contacts, shown as blue dashed lines.

The most prominent feature in the packing of (III) is the formation of a linear supra­molecular chain sustained by methine-C—H⋯O(carbon­yl) inter­actions, as illustrated in Fig. 2 ▸ *a*. The chains are aligned along the *c* axis and inter­actions between them are weak benzene-C—H⋯O(epoxide) and methine-C—H⋯O(meth­oxy) contacts to sustain a three-dimensional architecture, Fig. 2 ▸ *b*. Further insight into the mol­ecular packing is provided by an analysis of the Hirshfeld surface below.

## Hirshfeld surface analysis   

The Hirshfeld surfaces calculated on the structure of (III) was conducted in accord with a recent publication (Zukerman-Schpector *et al.*, 2017 ▸) and provides more insight into the inter­molecular inter­actions present in the crystal.

**Figure 3:**
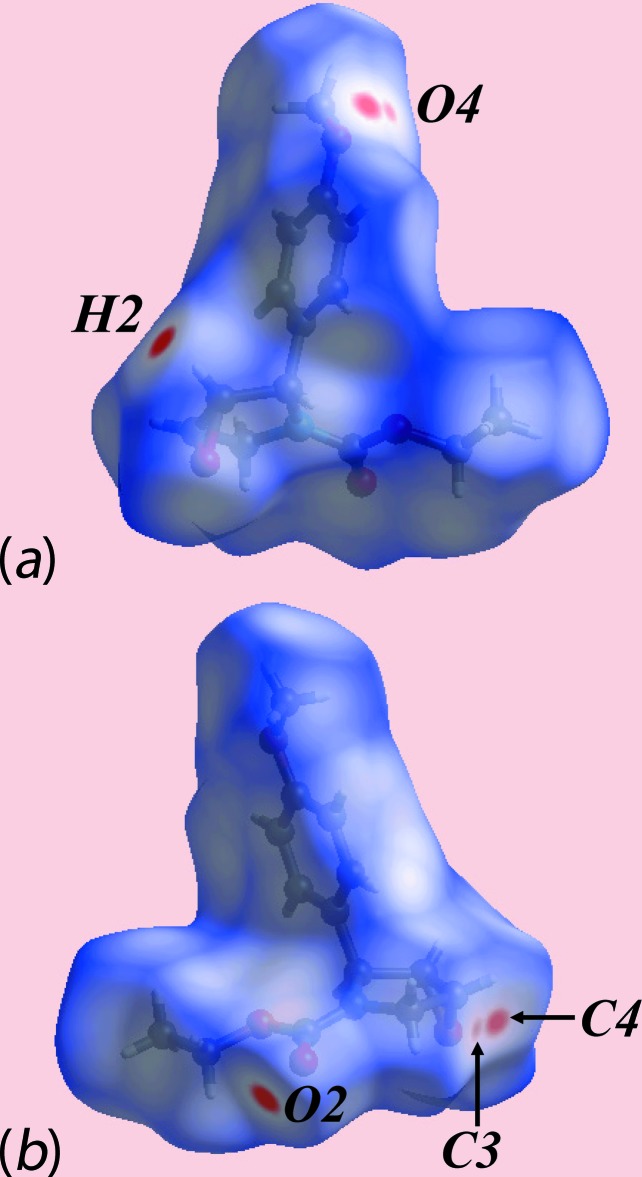
Two views of the Hirshfeld surface for (III) plotted over *d*_norm_ in the range −0.110 to 1.412 au.

**Figure 4:**
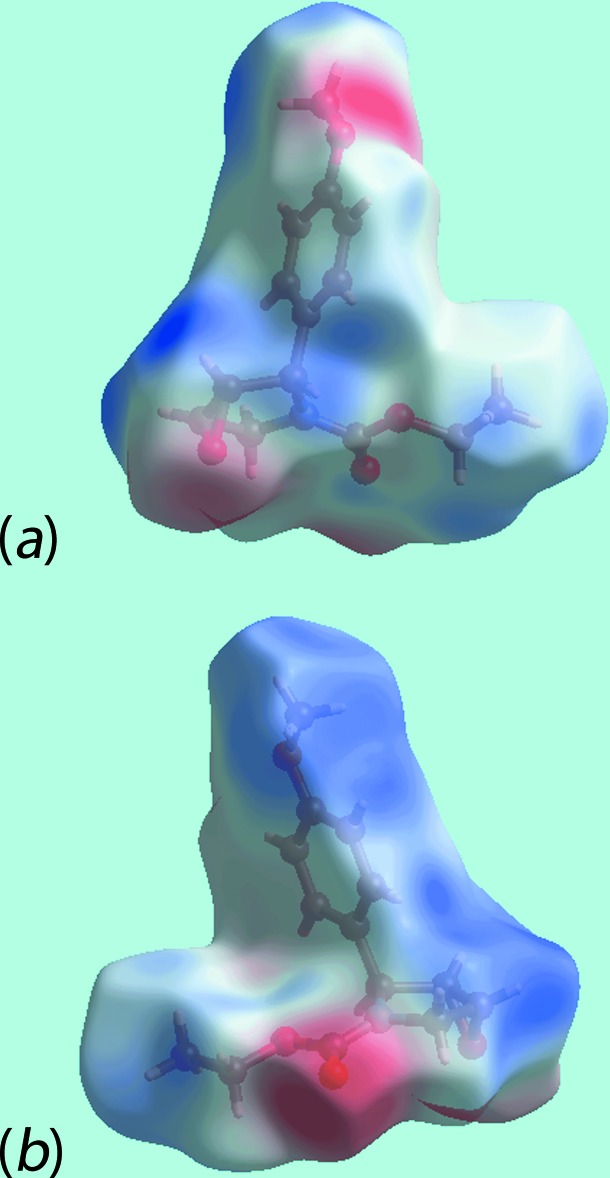
Two views of the Hirshfeld surface for (III) mapped over the calculated electrostatic potential in the range −0.083 to + 0.042 au. The red and blue regions represent negative and positive electrostatic potentials, respectively.

**Figure 5:**
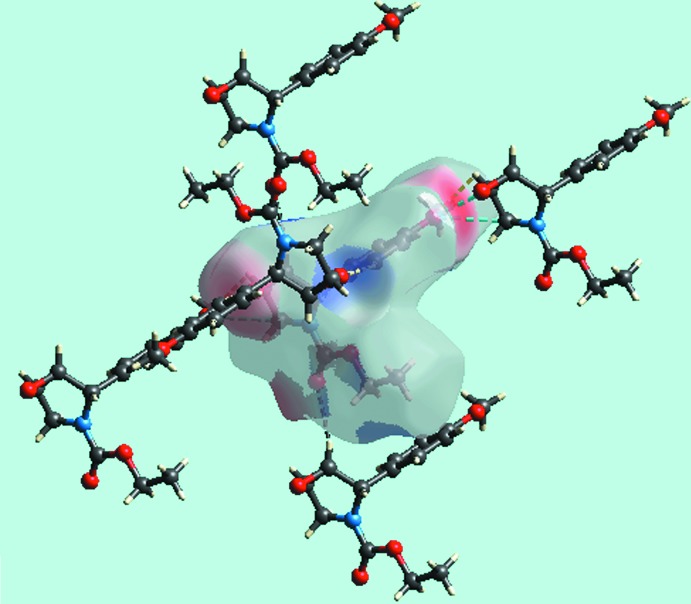
View of the Hirshfeld surface for (III) mapped over the electrostatic potential about a reference mol­ecule showing C—H⋯O, C⋯O/O⋯C and short inter-atomic O⋯H/H⋯O contacts with black, sky-blue and white dashed lines, respectively.

The donor and acceptor of the C—H⋯O hydrogen bond instrumental for the formation of the supra­molecular chain, *i.e*. between the methine-C—H2 and carboxyl­ate-O2 atoms, are viewed as the bright-red spots near these atoms on the Hirshfeld surface mapped over *d*_norm_ in Fig. 3 ▸ *a* and *b*. The bright-red spot, near meth­oxy-O4, and lighter spot, near methine-C3, and the diminutive red spot near meth­oxy-O4 and brighter spot near methyl­ene-C4 in Fig. 3 ▸, are indicative of another C—H⋯O inter­action (Table 1 ▸) and the short inter-atomic C⋯O/O⋯C contact (Table 2 ▸), respectively. On the Hirshfeld surface mapped over electrostatic potential in Fig. 4 ▸, the donors and acceptors of inter­molecular inter­actions are represented by blue and red regions, respectively, corresponding to positive and negative electrostatic potentials near the respective atoms. The immediate environment about a reference mol­ecule within Hirshfeld surfaces mapped over the electrostatic potential highlighting inter­molecular C—H⋯O inter­actions and short inter-atomic O⋯H/H⋯O contacts (Table 2 ▸) is illustrated in Fig. 5 ▸.

**Figure 6:**
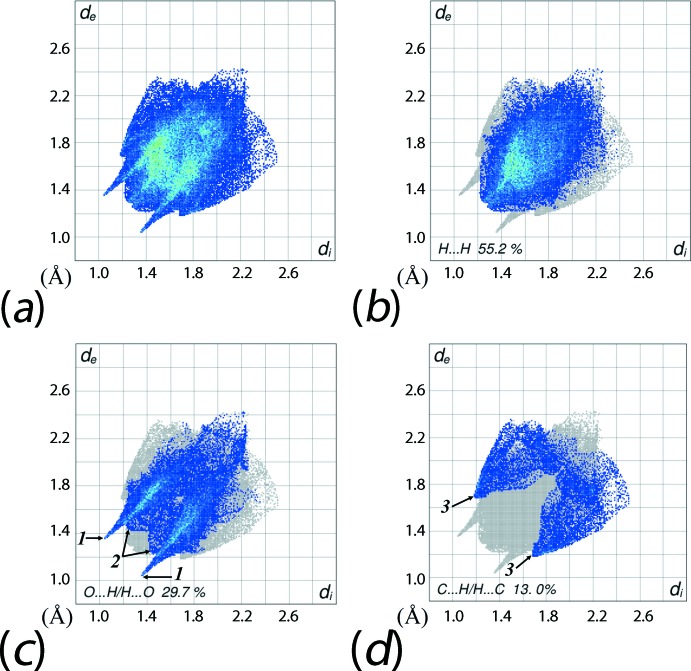
(*a*) The full two-dimensional fingerprint plot for (III) and fingerprint plots delineated into (*b*) H⋯H, (*c*) O⋯H/H⋯O and (*d*) C⋯H/H⋯C contacts.

The overall two dimensional fingerprint plot, Fig. 6 ▸ *a*, and those delineated into H⋯H, O⋯H/H⋯O and C⋯H/H⋯C contacts (McKinnon *et al.*, 2007 ▸) are illustrated in Fig. 6 ▸ *b*–*d*, respectively; the relative contributions from various contacts to the Hirshfeld surface are summarized in Table 3 ▸. The major contribution of 55.2% to the Hirshfeld surface is from inter-atomic H⋯H contacts, Fig. 6 ▸ *b*, and is indicative of dispersive forces operating in the crystal. In the fingerprint plot delineated into O⋯H/H⋯O contacts, Fig. 6 ▸ *c*, the 29.7% contribution results from the inter­molecular C—H⋯O inter­actions and short inter-atomic O⋯H/H⋯O contacts, Tables 1 ▸ and 2 ▸. In the plot, Fig. 6 ▸ *c*, a pair of spikes with their tips at *d*_e_ + *d*_i_ ∼2.4 Å (with label ‘1’) indicate the most significant C—H⋯O inter­action whereas the pair of two adjoining parabola with their peaks at around *d*_e_ + *d*_i_ ∼2.7 Å (label ‘2’) represent short inter-atomic O⋯H/H⋯O contacts. The presence of the short inter-atomic C⋯H/H⋯C contact, Table 2 ▸, hitherto not mentioned, in Fig. 6 ▸ *d*, leads to nearly symmetrical, characteristic wings with the pair of tips at *d*_e_ + *d*_i_ ∼2.9 Å as highlighted with label ‘3’. The low contributions from other contacts, Table 3 ▸, have a negligible effect on the packing as their inter-atomic distances are greater than sum of their respective van der Waals radii.

## Database survey   

There are three structures in the crystallographic literature (Groom *et al.*, 2016 ▸) having the basic framework shown at the top of Scheme 2, *i.e*. with non-specific bonds between the atoms. Each of the three structures retrieved from the search, *i.e*. (V), (VI) (Csatayová *et al.*, 2015 ▸) and (VII) (Rives *et al.*, 2010 ▸) in Scheme 2, has the same bonds in the framework. The common feature of (V)–(VII) is an envelope conformation for the pyrrolidyl ring with the flap atom being the N atom which is *syn* to the epoxide O1 atom, *i.e*. as for (III). Major conformational differences are evident, however. With reference to the pyrrolidyl ring, in (V) and (VI), in common with (III), the ring-bound substituents occupy positions opposite to that of the epoxide O atom but, in (VII), this substituent lies to the same side of the pyrrolidyl ring.
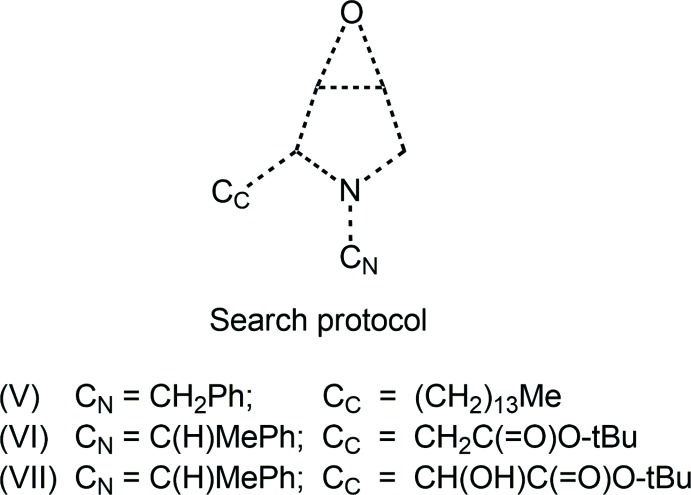


## Synthesis and crystallization   

The synthesis of (III) is as described in (Garcia, 2008 ▸). Crystals for the structure analysis were obtained by the slow evaporation of its CHCl_3_ solution. M. p. 378–379 K.

## Refinement details   

Crystal data, data collection and structure refinement details are summarized in Table 4 ▸. The carbon-bound H atoms were placed in calculated positions (C—H = 0.93–0.98 Å) and were included in the refinement in the riding model approximation, with *U*_iso_(H) set to 1.2–1.5*U*_equiv_(C).

## Supplementary Material

Crystal structure: contains datablock(s) I, global. DOI: 10.1107/S2056989017009987/hb7690sup1.cif

Structure factors: contains datablock(s) I. DOI: 10.1107/S2056989017009987/hb7690Isup2.hkl

Supporting information file. DOI: 10.1107/S2056989017009987/hb7690Isup3.cml

CCDC reference: 1560463

Additional supporting information:  crystallographic information; 3D view; checkCIF report

## Figures and Tables

**Table 1:** Hydrogen-bond geometry (Å, °)

*D*—H⋯*A*	*D*—H	H⋯*A*	*D*⋯*A*	*D*—H⋯*A*
C2—H2⋯O2^i^	0.98	2.40	3.3559 (19)	165
C13—H13⋯O1^ii^	0.93	2.68	3.456 (2)	155
C3—H3⋯O4^iii^	0.98	2.68	3.311 (2)	107

Symmetry codes: (i) 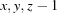; (ii) 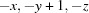; (iii) 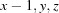.

**Table 2:** Summary of short inter-atomic contacts (Å) in (III)

Contact	Distance	Symmetry operation
C4⋯O4	3.167 (2)	−1 + *x*, *y*, *z*
O2⋯H9	2.63	*x*, 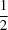 − *y*, − 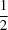 + *z*
C13⋯H6*A*	2.87	*x*, *y*, − 1 + *z*

**Table 3:** Percentage contributions of inter-atomic contacts to the Hirshfeld surfaces for (III)

Contact	Percentage contribution
H⋯H	55.2
O⋯H/H⋯O	29.7
C⋯H/H⋯C	13.0
C⋯C	1.1
N⋯H/H⋯N	0.5
C⋯O/O⋯C	0.4
O⋯O	0.1

**Table 4:** Experimental details

Crystal data	
Chemical formula	C_14_H_17_NO_4_
*M*_r_	263.28
Crystal system, space group	Monoclinic, *P*2_1_/*c*
Temperature (K)	293
*a*, *b*, *c* (Å)	9.6467 (9), 18.408 (1), 7.8076 (6)
β (°)	102.071 (8)
*V* (Å^3^)	1355.79 (18)
*Z*	4
Radiation type	Mo *K*α
μ (mm^−1^)	0.10
Crystal size (mm)	0.30 × 0.27 × 0.11

Data collection	
Diffractometer	Enraf–Nonius TurboCAD-4
Absorption correction	ψ scan (Blessing, 1995 ▸)
*T*_min_, *T*_max_	0.933, 0.990
No. of measured, independent and observed [*I* > 2σ(*I*)] reflections	4187, 3927, 2107
*R*_int_	0.028
(sin θ/λ)_max_ (Å^−1^)	0.703

Refinement	
*R*[*F* ^2^ > 2σ(*F* ^2^)], *wR*(*F* ^2^), *S*	0.049, 0.147, 1.00
No. of reflections	3927
No. of parameters	174
H-atom treatment	H-atom parameters constrained
Δρ_max_, Δρ_min_ (e Å^−3^)	0.17, −0.23

Computer programs: *CAD-4 EXPRESS* (Enraf–Nonius, 1989 ▸), *XCAD4* (Harms & Wocadlo, 1995 ▸), *SIR2014* (Burla *et al.*, 2015 ▸), *SHELXL2014* (Sheldrick, 2015 ▸), *ORTEP-3 for Windows* (Farrugia, 2012 ▸), *DIAMOND* (Brandenburg, 2006 ▸), *MarvinSketch* (ChemAxon, 2010 ▸) and *publCIF* (Westrip, 2010 ▸).

## supplementary crystallographic information

### Crystal data

**Table:**

C_14_H_17_NO_4_	*F*(000) = 560
*M**_r_* = 263.28	*D*_x_ = 1.290 Mg m^−^^3^
Monoclinic, *P*2_1_/*c*	Mo *K*α radiation, λ = 0.71073 Å
*a* = 9.6467 (9) Å	Cell parameters from 25 reflections
*b* = 18.408 (1) Å	θ = 11.0–13.6°
*c* = 7.8076 (6) Å	µ = 0.10 mm^−^^1^
β = 102.071 (8)°	*T* = 293 K
*V* = 1355.79 (18) Å^3^	Irregular, colourless
*Z* = 4	0.30 × 0.27 × 0.11 mm

### Data collection

**Table:**

Enraf–Nonius TurboCAD-4 diffractometer	*R*_int_ = 0.028
non–profiled ω/2τ scans	θ_max_ = 30.0°, θ_min_ = 2.2°
Absorption correction: ψ scan (Blessing, 1995)	*h* = −13→13
*T*_min_ = 0.933, *T*_max_ = 0.990	*k* = −25→0
4187 measured reflections	*l* = −10→0
3927 independent reflections	3 standard reflections every 60 min
2107 reflections with *I* > 2σ(*I*)	intensity decay: 1%

### Refinement

**Table:**

Refinement on *F*^2^	0 restraints
Least-squares matrix: full	Hydrogen site location: inferred from neighbouring sites
*R*[*F*^2^ > 2σ(*F*^2^)] = 0.049	H-atom parameters constrained
*wR*(*F*^2^) = 0.147	*w* = 1/[σ^2^(*F*_o_^2^) + (0.0677*P*)^2^ + 0.1423*P*] where *P* = (*F*_o_^2^ + 2*F*_c_^2^)/3
*S* = 1.00	(Δ/σ)_max_ < 0.001
3927 reflections	Δρ_max_ = 0.17 e Å^−^^3^
174 parameters	Δρ_min_ = −0.23 e Å^−^^3^

### Special details

**Table:**

Geometry. All esds (except the esd in the dihedral angle between two l.s. planes) are estimated using the full covariance matrix. The cell esds are taken into account individually in the estimation of esds in distances, angles and torsion angles; correlations between esds in cell parameters are only used when they are defined by crystal symmetry. An approximate (isotropic) treatment of cell esds is used for estimating esds involving l.s. planes.

### Fractional atomic coordinates and isotropic or equivalent isotropic displacement parameters (Å^2^)

**Table:**

	*x*	*y*	*z*	*U*_iso_*/*U*_eq_	
O1	−0.19247 (14)	0.42020 (8)	−0.03045 (18)	0.0678 (4)	
O2	−0.03454 (14)	0.36201 (7)	0.53678 (14)	0.0565 (3)	
O3	0.13930 (13)	0.42869 (6)	0.45731 (15)	0.0544 (3)	
O4	0.56099 (13)	0.25574 (8)	0.0389 (2)	0.0734 (4)	
N1	−0.01106 (14)	0.36816 (7)	0.25447 (16)	0.0467 (3)	
C1	0.05665 (17)	0.39804 (9)	0.1171 (2)	0.0456 (4)	
H1	0.0760	0.4499	0.1378	0.055*	
C2	−0.05889 (19)	0.38771 (10)	−0.0450 (2)	0.0518 (4)	
H2	−0.0341	0.3834	−0.1601	0.062*	
C3	−0.17188 (18)	0.34308 (11)	−0.0031 (2)	0.0548 (4)	
H3	−0.2228	0.3085	−0.0893	0.066*	
C4	−0.13558 (18)	0.32331 (10)	0.1864 (2)	0.0513 (4)	
H4A	−0.2129	0.3349	0.2438	0.062*	
H4B	−0.1134	0.2720	0.2019	0.062*	
C5	0.02590 (17)	0.38465 (9)	0.4251 (2)	0.0438 (4)	
C6	0.1888 (2)	0.45044 (11)	0.6374 (2)	0.0621 (5)	
H6A	0.2115	0.4081	0.7119	0.075*	
H6B	0.1163	0.4783	0.6776	0.075*	
C7	0.3173 (3)	0.49553 (15)	0.6432 (4)	0.0945 (8)	
H7A	0.3893	0.4668	0.6074	0.142*	
H7B	0.3517	0.5128	0.7605	0.142*	
H7C	0.2941	0.5362	0.5656	0.142*	
C8	0.19086 (17)	0.35884 (9)	0.10017 (19)	0.0439 (4)	
C9	0.19617 (19)	0.28404 (10)	0.0936 (3)	0.0601 (5)	
H9	0.1162	0.2574	0.1031	0.072*	
C10	0.31680 (19)	0.24737 (11)	0.0733 (3)	0.0605 (5)	
H10	0.3177	0.1969	0.0697	0.073*	
C11	0.43576 (17)	0.28635 (10)	0.0583 (2)	0.0528 (4)	
C12	0.43222 (19)	0.36132 (10)	0.0623 (3)	0.0594 (5)	
H12	0.5117	0.3878	0.0498	0.071*	
C13	0.31168 (18)	0.39740 (10)	0.0846 (2)	0.0529 (4)	
H13	0.3113	0.4479	0.0893	0.063*	
C14	0.5733 (2)	0.17891 (12)	0.0463 (3)	0.0757 (6)	
H14A	0.5536	0.1618	0.1548	0.114*	
H14B	0.6678	0.1651	0.0388	0.114*	
H14C	0.5069	0.1579	−0.0497	0.114*	

### Atomic displacement parameters (Å^2^)

**Table:**

	*U*^11^	*U*^22^	*U*^33^	*U*^12^	*U*^13^	*U*^23^
O1	0.0615 (8)	0.0775 (10)	0.0667 (8)	0.0171 (7)	0.0183 (6)	0.0127 (7)
O2	0.0670 (8)	0.0661 (8)	0.0413 (6)	−0.0002 (6)	0.0225 (5)	0.0039 (6)
O3	0.0607 (7)	0.0603 (7)	0.0425 (6)	−0.0093 (6)	0.0113 (5)	−0.0074 (5)
O4	0.0470 (7)	0.0723 (9)	0.1044 (11)	−0.0043 (7)	0.0235 (7)	−0.0145 (8)
N1	0.0513 (8)	0.0550 (8)	0.0367 (6)	−0.0106 (6)	0.0160 (5)	−0.0021 (6)
C1	0.0540 (9)	0.0467 (9)	0.0398 (8)	−0.0054 (7)	0.0184 (7)	0.0018 (7)
C2	0.0551 (10)	0.0630 (11)	0.0397 (8)	0.0063 (8)	0.0150 (7)	0.0046 (7)
C3	0.0493 (9)	0.0694 (12)	0.0464 (9)	0.0004 (8)	0.0111 (7)	−0.0061 (8)
C4	0.0492 (9)	0.0586 (10)	0.0490 (9)	−0.0081 (8)	0.0170 (7)	−0.0026 (8)
C5	0.0491 (9)	0.0430 (8)	0.0409 (8)	0.0047 (7)	0.0130 (7)	0.0008 (6)
C6	0.0721 (12)	0.0627 (12)	0.0483 (10)	−0.0001 (10)	0.0052 (9)	−0.0143 (9)
C7	0.0973 (19)	0.0905 (18)	0.0904 (17)	−0.0297 (14)	0.0075 (14)	−0.0280 (14)
C8	0.0470 (8)	0.0495 (9)	0.0368 (7)	−0.0076 (7)	0.0122 (6)	−0.0006 (7)
C9	0.0517 (10)	0.0525 (11)	0.0820 (13)	−0.0152 (8)	0.0277 (9)	−0.0069 (9)
C10	0.0560 (10)	0.0479 (10)	0.0829 (14)	−0.0100 (8)	0.0263 (9)	−0.0104 (9)
C11	0.0453 (9)	0.0609 (11)	0.0523 (9)	−0.0071 (8)	0.0103 (7)	−0.0104 (8)
C12	0.0443 (9)	0.0613 (11)	0.0734 (12)	−0.0166 (8)	0.0139 (8)	−0.0026 (9)
C13	0.0513 (10)	0.0486 (10)	0.0587 (10)	−0.0105 (8)	0.0111 (8)	0.0003 (8)
C14	0.0633 (12)	0.0743 (14)	0.0884 (15)	0.0086 (11)	0.0131 (11)	−0.0168 (12)

### Geometric parameters (Å, º)

**Table:**

O1—C3	1.443 (2)	C6—C7	1.485 (3)
O1—C2	1.447 (2)	C6—H6A	0.9700
O2—C5	1.2189 (18)	C6—H6B	0.9700
O3—C5	1.342 (2)	C7—H7A	0.9600
O3—C6	1.444 (2)	C7—H7B	0.9600
O4—C11	1.370 (2)	C7—H7C	0.9600
O4—C14	1.419 (3)	C8—C9	1.379 (2)
N1—C5	1.340 (2)	C8—C13	1.391 (2)
N1—C4	1.463 (2)	C9—C10	1.383 (2)
N1—C1	1.4739 (19)	C9—H9	0.9300
C1—C8	1.512 (2)	C10—C11	1.379 (2)
C1—C2	1.514 (2)	C10—H10	0.9300
C1—H1	0.9800	C11—C12	1.381 (3)
C2—C3	1.456 (2)	C12—C13	1.381 (3)
C2—H2	0.9800	C12—H12	0.9300
C3—C4	1.493 (2)	C13—H13	0.9300
C3—H3	0.9800	C14—H14A	0.9600
C4—H4A	0.9700	C14—H14B	0.9600
C4—H4B	0.9700	C14—H14C	0.9600
			
C3—O1—C2	60.50 (11)	C7—C6—H6A	110.4
C5—O3—C6	116.11 (13)	O3—C6—H6B	110.4
C11—O4—C14	118.28 (15)	C7—C6—H6B	110.4
C5—N1—C4	121.09 (13)	H6A—C6—H6B	108.6
C5—N1—C1	124.94 (14)	C6—C7—H7A	109.5
C4—N1—C1	113.68 (12)	C6—C7—H7B	109.5
N1—C1—C8	113.79 (13)	H7A—C7—H7B	109.5
N1—C1—C2	101.59 (13)	C6—C7—H7C	109.5
C8—C1—C2	111.17 (13)	H7A—C7—H7C	109.5
N1—C1—H1	110.0	H7B—C7—H7C	109.5
C8—C1—H1	110.0	C9—C8—C13	117.89 (16)
C2—C1—H1	110.0	C9—C8—C1	121.25 (14)
O1—C2—C3	59.63 (11)	C13—C8—C1	120.82 (15)
O1—C2—C1	113.23 (14)	C8—C9—C10	122.02 (16)
C3—C2—C1	109.74 (14)	C8—C9—H9	119.0
O1—C2—H2	119.9	C10—C9—H9	119.0
C3—C2—H2	119.9	C11—C10—C9	119.40 (18)
C1—C2—H2	119.9	C11—C10—H10	120.3
O1—C3—C2	59.87 (11)	C9—C10—H10	120.3
O1—C3—C4	112.52 (15)	O4—C11—C10	124.34 (17)
C2—C3—C4	109.22 (14)	O4—C11—C12	116.11 (15)
O1—C3—H3	120.2	C10—C11—C12	119.55 (17)
C2—C3—H3	120.2	C11—C12—C13	120.58 (16)
C4—C3—H3	120.2	C11—C12—H12	119.7
N1—C4—C3	103.12 (13)	C13—C12—H12	119.7
N1—C4—H4A	111.1	C12—C13—C8	120.55 (17)
C3—C4—H4A	111.1	C12—C13—H13	119.7
N1—C4—H4B	111.1	C8—C13—H13	119.7
C3—C4—H4B	111.1	O4—C14—H14A	109.5
H4A—C4—H4B	109.1	O4—C14—H14B	109.5
O2—C5—N1	124.47 (16)	H14A—C14—H14B	109.5
O2—C5—O3	124.44 (15)	O4—C14—H14C	109.5
N1—C5—O3	111.09 (13)	H14A—C14—H14C	109.5
O3—C6—C7	106.77 (17)	H14B—C14—H14C	109.5
O3—C6—H6A	110.4		
			
C5—N1—C1—C8	83.1 (2)	C1—N1—C5—O3	−4.0 (2)
C4—N1—C1—C8	−103.11 (16)	C6—O3—C5—O2	−1.0 (2)
C5—N1—C1—C2	−157.38 (15)	C6—O3—C5—N1	179.79 (14)
C4—N1—C1—C2	16.45 (18)	C5—O3—C6—C7	177.42 (17)
C3—O1—C2—C1	−100.05 (16)	N1—C1—C8—C9	47.4 (2)
N1—C1—C2—O1	54.73 (17)	C2—C1—C8—C9	−66.5 (2)
C8—C1—C2—O1	176.13 (13)	N1—C1—C8—C13	−134.82 (15)
N1—C1—C2—C3	−9.78 (18)	C2—C1—C8—C13	111.22 (17)
C8—C1—C2—C3	111.62 (15)	C13—C8—C9—C10	0.4 (3)
C2—O1—C3—C4	99.87 (16)	C1—C8—C9—C10	178.17 (16)
C1—C2—C3—O1	105.99 (15)	C8—C9—C10—C11	−0.3 (3)
O1—C2—C3—C4	−105.47 (16)	C14—O4—C11—C10	−4.4 (3)
C1—C2—C3—C4	0.5 (2)	C14—O4—C11—C12	175.67 (18)
C5—N1—C4—C3	157.71 (15)	C9—C10—C11—O4	179.54 (18)
C1—N1—C4—C3	−16.38 (18)	C9—C10—C11—C12	−0.6 (3)
O1—C3—C4—N1	−55.38 (17)	O4—C11—C12—C13	−178.80 (16)
C2—C3—C4—N1	9.10 (19)	C10—C11—C12—C13	1.3 (3)
C4—N1—C5—O2	3.4 (3)	C11—C12—C13—C8	−1.2 (3)
C1—N1—C5—O2	176.78 (15)	C9—C8—C13—C12	0.4 (3)
C4—N1—C5—O3	−177.35 (14)	C1—C8—C13—C12	−177.45 (15)

### Hydrogen-bond geometry (Å, º)

**Table:**

*D*—H···*A*	*D*—H	H···*A*	*D*···*A*	*D*—H···*A*
C2—H2···O2^i^	0.98	2.40	3.3559 (19)	165
C13—H13···O1^ii^	0.93	2.68	3.456 (2)	155
C3—H3···O4^iii^	0.98	2.68	3.311 (2)	107

Symmetry codes: (i) *x*, *y*, *z*−1; (ii) −*x*, −*y*+1, −*z*; (iii) *x*−1, *y*, *z*.
